# Editorial: Immunomodulatory Effects of Drugs for Treatment of Immune-Related Diseases

**DOI:** 10.3389/fimmu.2017.00969

**Published:** 2017-08-11

**Authors:** Azzam A. Maghazachi

**Affiliations:** ^1^Department of Clinical Medicine, College of Medicine and The Sharjah Institute for Medical Research, University of Sharjah, Sharjah, United Arab Emirates

**Keywords:** cancer, multiple sclerosis, chemokines, autophagy, adjuvants, natural killer cells

**Editorial on the Research Topic**

**Immunomodulatory Effects of Drugs for Treatment of Immune-Related Diseases**

The last 10 years have witnessed robust development of several drugs as a consequence of intense collaboration among academics, clinicians, scientists, and pharmaceutical companies. This has led to approval of these drugs for treatment of autoimmune diseases, cancer and AIDS, among many other diseases. This research topic focuses on the mechanism of action (MOA) of these drugs and reports new findings related to autophagy and adjuvanticity of drugs.

In an article by Kim et al., the function of the chemical 2-hydroxypropyl-β-cyclodextrin (HP-β-CD) was examined. This chemical has been previously used to facilitate the delivery of hydrophobic drugs and recently used as an adjuvant. The authors reported that HP-β-CD induced the maturation of dendritic cells (DCs), including upregulating the expression of co-stimulatory molecules and MHC class II molecules (Kim et al.). This maturation promoted these cells to become professional antigen-presenting cells inducing the proliferation of T cells. The adjuvanticity of the chemical was further examined in a mouse footpad immunization model, and it was concluded that HP-β-CD functions as a potent adjuvant inducing DCs maturation, a process mediated through lipid draft formation.

The theme of new adjuvants was further discussed in an article by Miccadei et al., who reviewed the potential effects of ω3-polyunsaturated fatty acids (PUFA) as an adjuvant for chemotherapy and/or radiotherapy regiments to treat colorectal cancer. Interest has been recently generated in the effects of diets on cancer, obesity, diabetes, and many more metabolic disorders. Administration of PUFA-enriched diets may have several advantages in controlling inflammatory responses that may benefit patients with inflammatory disorders. Further, PUFA-enriched diets might also be considered for preventing colorectal cancer, provided that the MOA of this adjuvant is clearly understood.

Musters et al. advocated the use of off-label drugs to treat immune-mediated diseases that failed all other possible treatments. The authors described a case of multicentric Castleman’s disease (MCD) with disseminated polyclonal B cell proliferation throughout multiple lymph nodes. This patient was treated in the authors’ center with tocilizumab, a humanized monoclonal antibody against human IL-6 receptor, which was approved for treatment of rheumatoid arthritis (RA) and juvenile RA patients, but not for Castleman’s disease patients. The patient showed a remarkable response to the drug including lower C-reactive protein level, improved sedimentation rate, and normalization of hemoglobin level. The authors concluded that off-label prescription drugs can be used after carefully evaluating the efficacy and safety of these biologic drugs.

The effects of latency-reversing agents (LRAs) on natural killer (NK) cells during clearance of HIV infection was explored in this issue (Garrido et al.). NK cells are the predominant innate immune cells that are important to fight against HIV infection (1), as well as many other viral infections. However, the effects of LRAs on NK cell activities have not been previously investigated in details, which are the subjects of this report (Garrido et al.). The authors utilized several drugs including histone deacetylase (HDAC) inhibitors such as SAHA or vorinostat, romidepsin, and panobinostat, which are standard chemotherapeutic agents for several cancer patients. They also used protein kinase C (PKC) agonists including prostratin and ingenol. Intriguingly, exposure to romidepsin and panobinostat was detrimental for NK cell activity, whereas exposure to the PKC agonists may be beneficial. Hence, caution should be exercised before these drugs can be considered to eradicate HIV infection, particularly in relation to their effects on the antiviral/antitumor effectors NK cells.

The potential effects of NK cells in cancer immunotherapy were also discussed (Maghazachi et al.). It was observed that NK cells incubated *in vitro* with two drugs; one approved for treatment of multiple sclerosis (MS), namely, dimethyl fumarate (DMF), and the second known as monomethyl fumarate (MMF), upregulated the expression of chemokine receptor 10 (CCR10) on NK cell surfaces. This was corroborated with increased chemotaxis of these cells toward the concentration gradients of the ligands for CCR10 such as CCL27 and CCL28, as well as enhanced NK cell cytotoxicity against tumor cell lines. These results might have clinical implication for harnessing the antitumor effector cells *in vitro* in order to induce their migration toward the sites of tumor growth, particularly those secreting CCL27 and CCL28. Further, the potential therapeutic effects of DMF and MMF for various disorders including MS, neurodegenerative diseases, cancer, and gastrointestinal ulcers were reviewed in this issue (Al-Jaderi and Maghazachi). The authors described the effects of DMF and MMF on various immune cells including T cells, B cells, NK cells, DCs, macrophages, and neutrophils, as well as on keratinocytes, endothelial cells, microglia, astrocytes, and neurons (Al-Jaderi and Maghazachi).

The topic of autophagy was discussed in two separate publications. Poon et al. described a novel effect of collagen type V alpha 1 (COL5A1) in exacerbating asthma. The authors suggested that autophagy-related 5 (ATG5) gene expression was positively correlated with the expression of COL5A1, and the subsequent deposition of collagen in the lungs of refractory asthmatic patients. These results indicate that dysregulation of autophagy may correlate with the fibrosis in the airway of asthmatic patients, suggesting that ATG5 might be a target for therapy in refractory asthmatic patients. On the other hand, Li et al. described beneficial effects for autophagy in autoimmune hepatitis, an autoimmune disorder characterized by abnormal Th1 activation and reduced apoptosis. It was observed that mice rich in n-3 PUFA resist liver damage after the administration of concanavalin-A, which induced hepatitis in these mice. The authors sought to examine the MOA of n-3 PUFA and observed that this fatty acid enhanced T cell autophagy and reduced T cell activation resulting in protection against severe liver injury. Collectively, these observations might form a basis for the potential therapeutic application of n-3 PUFA as well as other related fish products such as eicosapentaenoic acid or docosahexaenoic acid, toward liver damage and autoimmune hepatitis.

Hematological disorders were described in two papers by the same authors. In the first, Brenner et al. studied the effects of chemokine receptor expression in 79 patients with acute myeloid leukemia (AML) an aggressive form of leukemia and reported that CCL28 is constitutively released by primary human AML cells. Therefore, this chemokine may play a role in the cross talk among leukemic cells and neighboring bone marrow cells. However, the effect can be either inhibitory for the growth of leukemia if stem cell factor or GM-CSF factor is present, whereas the final effect of CCL28 can be stimulatory when Flt3 ligand is exogenously released. Although it was suggested that blocking chemokine receptor in this model might be beneficial for AML patients, caution should be exercised as CCR10, the receptor for CCL28, has been found to be up-regulated on the surface of the anti-tumor NK cells, which may facilitate their recruitment toward the sites of tumor growth (Maghazachi et al.). Therefore, a balance between the stimulatory and inhibitory effects of chemokine/chemokine receptor expression must be carefully weighed before regiments of cancer therapy are developed based on this concept. In a subsequent study, the same authors examined cytokine-mediated cross talk among AML and mesenchymal stem cells (MSCs), and observed that MSCs release cytokines that increase the viability and proliferation of leukemic cells, despite the heterogeneity of AML (Brenner et al.). These findings may have implications in targeting the relevant cytokines or their receptors for therapeutic purposes of aggressive leukemia.

In summary, the topics described in this issue should form the basis for understanding the MOA of drugs that may facilitate the development of personalized medicine to treat solid tumors, blood lymphoproliferative disorders, autoimmune diseases, and inflammatory conditions.

## Author Contributions

The author confirms being the sole contributor of this work and approved it for publication.

## Conflict of Interest Statement

The author declares that the research was conducted in the absence of any commercial or financial relationships that could be construed as a potential conflict of interest.
